# A survey of Australian chiropractors’ attitudes and beliefs about evidence-based practice and their use of research literature and clinical practice guidelines

**DOI:** 10.1186/2045-709X-21-44

**Published:** 2013-12-17

**Authors:** Bruce F Walker, Norman J Stomski, Jeff J Hebert, Simon D French

**Affiliations:** 1 School of Health Professions, Murdoch University, 90 South St., Murdoch, WA 6150, Australia; 2 School of Psychology and Exercise Science, Murdoch University, 90 South St, Murdoch, WA 6150, Australia; 3 School of Rehabilitation Therapy, Queen’s University, Kingston, ONT, Canada; 4 Centre for Health, Exercise and Sports Medicine, University of Melbourne, Grattan Street, Parkville, VIC 3010, Australia

## Abstract

**Background:**

Research into chiropractors’ use of evidence in clinical practice appears limited to a single small qualitative study. The paucity of research in this area suggests that it is timely to undertake a more extensive study to build a more detailed understanding of the factors that influence chiropractors’ adoption of evidence-based practice (EBP) principles. This study aimed to identify Australian chiropractors’ attitudes and beliefs towards EBP in clinical practice, and also examine their use of research literature and clinical practice guidelines.

**Methods:**

We used an online questionnaire about attitudes, beliefs and behaviours towards the use of EBP in clinical practice that had been developed to survey physiotherapists and modified it to ensure that it was relevant to chiropractic practice. We endeavoured to survey all registered Australian chiropractors (n = 4378) via email invitation distributed by Australian chiropractic professional organisations and the Chiropractic Board of Australia. Logistic regression analyses were conducted to examine univariate associations between responses to items measuring attitudes and beliefs with items measuring: age; years since registration; attention to literature; and use of clinical practice guidelines.

**Results:**

Questionnaires were returned by 584 respondents (response rate approximately 13%). The respondents’ perceptions of EBP were generally positive: most agreed that the application of EBP is necessary (77.9%), literature and research findings are useful (80.2%), EBP helps them make decisions about patient care (66.5%), and expressed an interest in learning or improving EBP skills (74.9%). Almost half of the respondents (45.1%) read between two to five articles a month. Close to half of the respondents (44.7%) used literature in the process of clinical decision making two to five times each month. About half of the respondents (52.4%) agreed that they used clinical practice guidelines, and around half (54.4%) agreed that they were able to incorporate patient preferences with clinical practice guidelines. The most common factor associated with increased research uptake was the perception that EBP helps make decisions about patient care.

**Conclusions:**

Most Australian chiropractors hold positive attitudes towards EBP, thought EBP was useful, and were interested in improving EBP skills. However, despite the favourable inclination towards EBP, many Australian chiropractors did not use clinical practice guidelines. Our findings should be interpreted cautiously due to the low response rate.

## Background

Evidence based practice (EBP) involves the “integration of best research evidence with clinical expertise and patient values and circumstances” [1]. The aim of EBP is to provide practitioners with an efficient means of keeping abreast of the ever-increasing amount of healthcare literature [2]. Proponents of EBP believe that practitioners can use best evidence as an integral part of their clinical decision-making [2].

There has been a growing emphasis on EBP over the last two decades. The increased awareness about the importance of EBP may be due to higher rates of litigation, demands for greater accountability for clinical practice, and increased public awareness of healthcare associated with improved information technology [2-4]. While much remains to be undertaken to improve healthcare professionals’ use of EBP principles, there have been substantial improvements in the adoption of EBP for most health disciplines [5-7].

Clinician adherence to EBP principles may result in numerous clinical benefits including: improved quality of healthcare delivery; enhanced professional credibility; facilitation of interdisciplinary collaboration; standardisation of care; and less reliance on non-credible or unreliable information sources to inform clinical decision-making [2,4,8-11]. Such benefits suggest that clinicians have an ethical and professional obligation to adopt a clinical approach consistent with EBP principles to ensure that their patients receive the most effective and safest possible healthcare.

In order for evidence to be incorporated into clinical practice, there must be a number of factors present. Practitioners initially require motivation and genuine desire to utilise research evidence [12]. Next, practitioners need to be able to identify gaps in their own knowledge and then frame appropriate questions [12]. The practitioners then need to develop skills to conduct an efficient literature search; critically appraise the research evidence; apply the literature findings appropriately to the patient problem; and then evaluate the application of evidence.

Although much has been written about EBP in chiropractic journals and by chiropractic authors in recent years [13-23], research investigating chiropractors’ use of EBP principles appears limited to a single small qualitative study [24]. That study of seven practising chiropractors found that they tended to view EBP more as an academic exercise and questioned its relevance to clinical practice. Moreover, the chiropractors did not routinely use EBP principles and lacked the ability and confidence to appraise research evidence [24]. These findings, and the paucity of research in this area, suggest that it is timely for the chiropractic profession to undertake a more extensive study to build a more detailed understanding of factors that influence chiropractors’ adoption of EBP principles. Therefore, this study aimed to identify through the use of a structured electronic questionnaire Australian chiropractors’ attitudes and beliefs towards EBP in clinical practice, and also examine their use of research literature and clinical practice guidelines.

## Methods

### Questionnaire development

We used a modified version of the Jette et al. questionnaire, which was developed to survey physiotherapists and was found to be a reliable instrument [25]. The original questionnaire contained seven scales, which in total comprised 32 items that captured information about:

• attitudes and beliefs about EBP

• interest and motivation to engage in EBP

• educational background and knowledge and skills related to accessing and interpreting information

• attention to literature, which involved reading research literature, using research literature in making clinical decisions, and the use of medical databases

• availability and ability to access information

• access and use of practice guidelines

• perceived barriers to using evidence in practice

The Jette et al. questionnaire was adapted for this study by using a Content Validity Index (CVI) to ensure that it was relevant to use among chiropractors. Deriving a CVI involved the participation of an expert panel of nine chiropractors (six clinicians, two educators, and one researcher). The composition and size of this panel accords with recommendations that suggest that the members’ professional backgrounds should reflect that of the target population, and that the ideal number ranges from six to twelve [26,27]. Each panel member assessed each item using four categories: not relevant, unable to assess relevance without major revision, relevant but needs minor alteration, and very relevant. A value of one was assigned to the “very relevant” and “relevant but needs minor alteration” categories, whereas a value of zero was assigned to the other categories. The CVI for each item was derived by summing the values for each rater and dividing by the number of raters. Items were retained if the CVI exceeded 0.79 [26,27]. Twenty seven of the 32 items achieved a CVI greater than 0.79 (range 0.88–1.0). The five omitted items which recorded a CVI below 0.79 were: I need to increase the use of evidence in my daily practice; EBP does not take into account the limitations of my clinical practice setting; My reimbursement rate will increase if I incorporate EBP into my practice; EBP does not take into account patient preference; My practice supports the use of current research in practice (Additional file 1).

### Sample accrual

The target population was all registered Australian chiropractors (n = 4378). We did not anticipate a high response rate but instead a large enough sample to be indicative within the constraints that non-random samples provide.

### Survey implementation

An initial invitation and link to the online survey, followed by two fortnightly reminders were distributed via email by the Chiropractic and Osteopathic College of Australasia, and then the Chiropractors Association of Australia. Finally, the Chiropractic Board of Australia also placed the invitation and survey link in its electronic newsletter which is distributed to registered chiropractors who have provided their email address. This procedure was administered over a 3 month period of early 2013. Participants were directed to read an information letter embedded at the beginning of the online survey and provide their consent to participate prior to completing the questionnaire. Responses were automatically downloaded into SPSS v.21 and remained anonymous.

### Ethics

Ethics approval was received from the Murdoch University Human Research Ethics Committee (approval number 2013/007) and consent was implied by participants who completed the survey.

### Data analysis

Data were analysed using SPSS v.21. Subsequent to the implementation of the survey and completing data collection, we examined the dimensionality of the predefined response option items in the adapted questionnaire by using principal component analysis with varimax rotation, which identified five strongly loading factors:

• Factor 1 (items 1–5 and item 7) related to a construct reflecting attitudes and beliefs towards EBP

• Factor 2 (items 8–10) related to a construct reflecting attention to literature

• Factor 3 (items 11–16) related to a construct reflecting use of clinical practice guidelines

• Factor 4 (items 17–19) related to a construct reflecting access to research evidence

• Factor 5 (items 20–25) related to a construct reflecting EBP knowledge and skills.

Item 6 did not load strongly onto any factor and was not included in further analyses.

Items were grouped into scales according to the factors they strongly loaded onto and the scales’ homogeneity by using corrected item-total correlations and Cronbach's alpha were examined [28]. The corrected item-total correlations ranged from 0.39-0.79 and the Cronbach's alpha values for the “attitudes and beliefs”, “attention to literature”, “use of clinical practice guidelines”, “access to research evidence”, and “EBP knowledge and skills” scales were respectively 0.89, 0.80, 0.77, 0.73, and 0.79, which indicated acceptable internal consistency [28].

All data were reported descriptively. To partially analyse non-response bias age was classified into categories used by the Chiropractic Board of Australia so the age characteristics of the responders could be compared to the entire Australian chiropractic population. Before examining associations between variables, we considered response distributions and collapsed some categories for use as dependent variables in logistic regression analyses. For 5-point Likert scale items with positive wording (ie agreement with the statement suggested positive regard for EBP), the “strongly agree” and “agree’” categories were combined, as were the “neutral”, “disagree” and “strongly disagree” categories”, leaving 1 of 2 categories: “agree” or “disagree”. For 5-point Likert scale items with negative wording, the “neutral category” was combined with the “agree” and “strongly agree” categories. For items with “yes”, “no” and “do not know” response options, “no” was combined with “do not know”, as it seems likely, for instance, that not knowing whether internet access was available was as unhelpful as not having internet access. For items capturing information about the number of times articles were read or databases accessed per month, the lowest category, namely one or no articles per month, was distinguished from higher categories given that it reflects poor attention to the literature which would be inconsistent with EBP principles.

Logistic regression analyses were conducted to examine the following univariate associations: (1) responses to items measuring attitudes and beliefs with items measuring; age; years since registration; use of clinical practice guidelines. Odds ratios and their 95% confidence intervals were calculated. Odds ratios in this study described the likelihood of demonstrating a particular behaviour (e.g., reading research evidence) given an individual characteristic (e.g., interest in EBP).

## Results

In this paper we report findings from the attitudes and beliefs, attention to literature, and use of clinical guidelines sections of the questionnaire. The results from the other sections will be detailed in a subsequent article.

Questionnaires were returned by 584 respondents (response rate approximately 13%). Some questionnaires were incomplete, which explains the discrepancy in response numbers between individual items.

### Demographic characteristics

Demographic details are displayed in Table 1. Respondents were predominantly male (73.4%). About one third of the respondents were aged 31–40 (31.3%). Most respondents practiced in a metropolitan location (63.5%). Respondents most commonly worked 30 to 40 (37.7%) hours per week.

**Table 1:** Respondent demographic characteristics

	**Mean**	**Standard deviation**	**National %**
**Years since registration**	15.7	11.0	
**Gender**	**No. (n = 515)**	**%**	
Females	136	26.4	
**Age**	**No. (n = 515)**	%	
21-30	91	17.9	20.6
31-40	161	31.3	31.7
41-50	120	23.3	23.4
51-60	103	20.0	14.6
61 and above	39	7.6	9.7
**State/territory**	**No. (n = 519)**	**%**	
Australian capital territory	13	2.5	1.3
New South Wales	141	27.1	33.4
Northern territory	4	1.0	0.5
Queensland	81	15.6	15.3
South Australia	53	10.5	7.8
Tasmania	5	1.0	1.0
Victoria	133	25.6	26.9
Western Australia	89	17.1	11.3
**Practice location**			
Rural	63	12.3	
Regional	124	24.2	
Suburban	325	63.5	
**Membership**			
Chiropractors’ Association of Australia	365		
Chiropractic and Osteopathic College of Australasia	196		
Australian Spinal Research Foundation	144		
**Average work hours per week**			
Less than twenty	99	19.3	
Twenty to thirty	146	28.5	
Thirty to forty	193	37.7	
More than forty	74	14.5	
**Average number of patients seen per typical day**			
Five or less	41	8.0	
Six to ten	62	12.1	
Eleven to fifteen	65	12.6	
Sixteen to twenty	86	16,7	
Twenty one to thirty	111	21.6	
Thirty one to forty	70	13.6	
More than forty	79	15.4	

• Questionnaires were returned by 584 respondents. Some questionnaires were incomplete, hence the discrepancy in response numbers between items.

### Attitudes and beliefs

Responses to items enquiring about attitudes and beliefs are displayed in Table 2. The respondents’ perceptions of EBP were generally positive: most agreed that EBP is necessary (77.9%), literature and research findings are useful (80.2%), EBP assists in decisions about patient care (66.5%), and expressed interest in learning or improving EBP skills (74.9%). About half of respondents (56.6%) disagreed that using EBP places an unreasonable demand on chiropractors.

**Table 2:** Self-reported attitudes and beliefs

	**Disagree**	**Neutral**	**Agree**
Application of EBP is necessary in the practice of chiropractic (n = 583)	11.0%	11.1%	77.9%
Literature and research findings are useful in my day-to-day practice (n = 582)	9.1%	10.7%	80.2%
The adoption of EBP places an unreasonable demand on chiropractors (n = 583)	55.6%	23.7%	20.8%
I am interested in learning or improving the skills necessary to incorporate EBP into my practice (n = 582)	7.9%	17.2%	74.9%
EBP improves the quality of patient care (n = 579)	20.1%	18.7%	61.4%
EBP helps me make decisions about patient care (n = 582)	16.2%	17.4%	66.5%

Demographic factors were weakly associated with some aspects of EBP attitudes and beliefs (Tables 3 and 4). In cases where associations were observed, older chiropractors were less likely to agree that EBP is necessary (OR 0.96; 95% CI 0.95-0.98), but less likely to agree that the adoption of EBP places an unreasonable demand on chiropractors (OR 0.98; 95% CI 0.97-0.99). Also, chiropractors who had been registered for more years were less likely to agree that the application of EBP is necessary (OR 0.96; 95% CI 0.95-0.98) and less likely to express an interest in learning or improving EBP skills (OR 0.98; 95% CI 0.96-0.97), but less likely to agree that the adoption of EBP places an unreasonable demand on chiropractors (OR 0.98; 95% CI 0.96-0.99).

**Table 3:** Associations between age and self-reported attitudes and beliefs

	**OR**	**95% CI**
Application of EBP is necessary in the practice of chiropractic	0.96*	0.95 0.98
Literature and research findings are useful in my day-to-day practice	1.01	0.99-1.02
The adoption of EBP places an unreasonable demand on chiropractors	0.98*	0.97-0.99
I am interested in learning or improving the skills necessary to incorporate EBP into my practice.	0.99	0.97-1.00
EBP improves the quality of patient care	0.99	0.98-1.01
Strong evidence is lacking to support most of the interventions I use with my patients	1.00	0.99-1.02
EBP helps me make decisions about patient care	0.99	0.98-1.01

*Significant finding.

**Table 4:** Associations between years since registration and self-reported attitudes and beliefs

	**OR**	**95% CI**
Application of EBP is necessary in the practice of chiropractic	0.96*	0.95-0.98
Literature and research findings are useful in my day-to-day practice	0.99	0.98-1.02
The adoption of EBP places an unreasonable demand on chiropractors	0.98*	0.96-0.99
I am interested in learning or improving the skills necessary to incorporate EBP into my practice.	0.98*	0.96-0.99
EBP improves the quality of patient care	0.99	0.97-1.01
Strong evidence is lacking to support most of the interventions I use with my patients	1.00	0.99-1.02
EBP helps me make decisions about patient care	0.99	0.97-1.00

*Significant finding.

### Attention to literature

Responses to items enquiring about attention to research literature are displayed in Table 5. Almost half of the respondents (45.1%) read two to five articles a month. Slightly less than half of the respondents (45.3%) searched medical databases once or not at all each month. Close to half of the respondents (44.7%) used literature to inform clinical decision-making two to five times each month.

**Table 5:** Self-reported attention to literature

**Times per month**					
	**None or once**	**2-5**	**6-10**	**11-15**	**16 or more**
Read/review research/literature related to my clinical practice (n = 581)	16.5%	45.1%	20.1%	8.1%	10.2%
I use professional literature and research findings in the process of clinical decision-making (n = 579)	21.2%	44.7%	15.4%	5.5%	13.1%
I use The Cochrane Library, MEDLINE, PUBMED or other databases to search for practice-relevant literature/research (n = 579)	45.3%	30.6%	11.9%	4.7%	7.6%

In considering the attitude and belief items, attention to research literature was associated with believing that literature was useful, and that EBP helps make decisions about patient care (Tables 6, 7 and 8). Respondents perceiving that literature was useful were three times more likely to read literature related to clinical practice at least twice per month (OR 2.91; 95% CI 1.60-5.29) and nearly five times more likely to use literature in clinical decision-making at least twice per month ( OR 4.62; 95% CI 2.67-7.99). Respondents perceiving that EBP helps make decisions about patient care were twice as likely to read literature at least twice a month (OR 1.93; 95% CI 1.05-3.56), three times more likely to use literature in clinical decision making at least twice per month (OR 3.0; OR 95% CI 1.70-5.30), and about one and a half times more likely to use a medical database at least twice per month (OR 1.68; 95% CI 1.06-2.68).

**Table 6:** Associations between self-reported attitudes and beliefs and reading at least two research articles per month

	**OR**	**95% CI**
Application of EBP is necessary in the practice of chiropractic	0.88	0.46-1.66
Literature and research findings are useful in my day-to-day practice	2.91*	1.60-5.29
The adoption of EBP places an unreasonable demand on chiropractors	1.78*	1.00-3.17
I am interested in learning or improving the skills necessary to incorporate EBP into my practice.	1.73	0.95-3.17
EBP improves the quality of patient care	0.44	1.23-0.86
EBP helps me make decisions about patient care	1.94*	1.05-3.56

*Significant finding.

**Table 7:** Associations between self-reported attitudes and beliefs and using literature in clinical decision making at least twice per month

	**OR**	**95% CI**
Application of EBP is necessary in the practice of chiropractic	0.82	0.45-1.50
Literature and research findings are useful in my day-to-day practice	4.62*	2.67-7.80
The adoption of EBP places an unreasonable demand on chiropractors	1.66	0.94-2.93
I am interested in learning or improving the skills necessary to incorporate EBP into my practice.	1.56	0.89-2.75
EBP improves the quality of patient care	1.06	0.58-1.93
EBP helps me make decisions about patient care	2.99*	1.70-5.30

*Significant finding.

**Table 8:** Associations between self-reported attitudes and beliefs and using medical databases at least twice per month

	**OR**	**95% CI**
Application of EBP is necessary in the practice of chiropractic	1.23	0.73-2.08
Literature and research findings are useful in my day-to-day practice	1.54	0.91-2.60
The adoption of EBP places an unreasonable demand on chiropractors	1.46	0.96-2.22
I am interested in learning or improving the skills necessary to incorporate EBP into my practice.	1.59	0.98-2.56
EBP improves the quality of patient care	1.10	0.70-1.74
EBP helps me make decisions about patient care	1.68*	1.00-2.68

*Significant finding.

### Use of clinical practice guidelines

Responses to items enquiring about the use of clinical practice guidelines are displayed in Table 9. Slightly more than half of the respondents (57.7%) were aware that practice guidelines are available online, less than half (42.3%) actively sought practice guidelines, and almost three quarters (72.3%) were able to access online guidelines. About half of the respondents used clinical practice guidelines (52.4%), and were able to incorporate patient preferences (54.4%).

**Table 9:** Self-reported use of clinical guidelines

	**Yes**	**No**	
I am aware that practice guidelines are available online (n = 560)	57.7%	42.3%	
I am able to access practice guidelines online (n = 555)	72.3%	27.7%	
	**Disagree**	**Neutral**	**Agree**
I actively seek practice guidelines pertaining to areas of my practice (n = 561)	26.4%	31.4%	42.3%
I use practice guidelines in my practice (n = 562)	16%	31.5%	52.4%
I am able to incorporate patient preferences with practice guidelines (n = 560)	7.2%	38.4%	54.4%

The only attitudes and beliefs item associated with the seeking and use of clinical practice guidelines was the statement that EBP helps make decisions about patient care (Tables 10 and 11). Respondents who perceived that EBP helps make decisions about patient care were about one and a half times more likely to seek practice guidelines (OR 1.66; 95% CI 1.01-2.74), almost three times more likely to use clinical practice guidelines (OR 2.80; 95% CI 1.73-4.54), and three times more likely to agree that patients preferences could be incorporated with clinical practice guidelines (OR 2.93; 95% CI 1.81-4.74).

**Table 10:** Associations between self-reported attitudes and beliefs and seeking clinical practice guidelines more than per month

	**OR**	**95% CI**
Application of EBP is necessary in the practice of chiropractic	1.16	0.66-2.07
Literature and research findings are useful in my day-to-day practice	1.83	0.99-3.33
The adoption of EBP places an unreasonable demand on chiropractors	1.38	0.89-2.13
I am interested in learning or improving the skills necessary to incorporate EBP into my practice.	1.73*	1.03-2.91
EBP improves the quality of patient care	1.07	0.67-1.71
EBP helps me make decisions about patient care	1.66*	1.01-2.74

*Significant finding.

**Table 11:** Associations between self-reported attitudes and beliefs and using clinical practice guidelines to inform decision making at least twice per month

	**OR**	**95% CI**
Application of EBP is necessary in the practice of chiropractic	1.39	0.81-2.40
Literature and research findings are useful in my day-to-day practice	1.07	0.62-1.86
The adoption of EBP places an unreasonable demand on chiropractors	1.22	0.79-1.88
I am interested in learning or improving the skills necessary to incorporate EBP into my practice.	1.43	0.87-2.35
EBP improves the quality of patient care	0.98	0.61-1.56
EBP helps me make decisions about patient care	2.80*	1.73-4.54

*Significant finding.

## Discussion

Our findings suggest that most Australian chiropractors who responded to our survey viewed EBP as an essential component of chiropractic practice. Respondents thought research literature was useful in day-to-day practice, and indicated that EBP improved the quality of their patient care and assists in decision making. Respondents also expressed an interest in further developing EBP skills. These findings were consistent with results in studies of nurses, general practitioners, physiotherapists, occupational therapists, speech-language therapists and complementary therapists [9,29-38].

The association in our study between the respondents’ age and time since registration and their attitudes and beliefs was in line with several previous studies, in which most respondents report positive attitudes and beliefs irrespective of age and clinical practice years [29,30,33-37]. However, a few previous studies have shown that older, more clinically experienced respondents were less likely to demonstrate positive attitudes and beliefs towards EBP [31,32]. These findings are consistent with only two of our results which showed that older respondents and those who had been registered for more time were less likely to agree that EBP was necessary, although these factors influenced their views about the necessity of EBP only to a marginal extant.

Across previous studies of health professionals, holding a higher educational qualification, particularly a Masters level or above, is the only factor that has been consistently associated with positive beliefs and attitudes towards EBP [30,31,33-35,39,40]. We did not enquire about educational qualifications and therefore it is unclear if this factor influences Australian chiropractors’ attitudes towards EBP. However, examining the influence of higher educational qualifications would have been difficult in the context of our study as many Australian trained chiropractors receive coursework Masters Degrees or equivalent upon completing their educational training.

The respondents in our study most commonly read between two to five research articles per month. This level was congruent with the reported rates in studies of orthodontists, occupational therapists, and physiotherapists [31,34,40]; higher than one study involving physiotherapists [25]; and lower than another study of physiotherapists [41]. Although the number of articles the respondents in our study most commonly read each month may seem low, it may not necessarily be inconsistent with EBP principles. In particular, if clinicians are managing patients with a small range of conditions there may be relatively little need to read widely. A recent study of Australian chiropractic practice found that chiropractors reported that over 90% of consultations addressed musculoskeletal problems [42]. However, it is also the case that there is large amount of new musculoskeletal research published on a frequent basis, and one could argue that clinicians need to read widely to remain up to date with the best available evidence. It seems doubtful, though, that most clinicians would be well equipped to synthesise the constant stream of new research findings and then use it to modify recommendations contained in systematic reviews and clinical practice guidelines. Given this, perhaps it is time to amend the old EBP paradigm and refocus the roles of clinicians by placing the emphasis on remaining current with evidence summaries, systematic reviews, and clinical practice guidelines.

The infrequent use of medical databases may also possibly be consistent with EBP principles. The main chiropractic journals tend to publish articles on a monthly basis and accessing databases once each month would be sufficient to keep abreast of relevant literature. Nonetheless, the respondents’ attention to research literature was significantly increased when they perceived that literature was useful and that EBP helps them make decisions about patient care. These findings suggest that chiropractic professional development programs should highlight the importance of research literature and its usefulness in improving clinical decision making.

Although most respondents viewed EBP as an essential component of chiropractic practice, only about half of the respondents in our study used clinical practice guidelines. This finding is of concern but is consistent with one previous study of physiotherapists [30] yet lower than that reported in another study of physiotherapists (over 90% used clinical practice guidelines) [34], lower than a study of allied health professionals (75% used clinical practice guidelines) [36], and lower than a study of health professionals in public hospitals (about 66% used clinical practice guidelines) [43]. The respondents in our study were significantly more likely to use clinical practice guidelines when they perceived that EBP assisted in decisions about patient care. This finding suggests that to increase chiropractors’ uptake of EBP educational interventions should highlight improvements in patient outcomes that may result from clinical practice guideline adherence.

Why the positive attitudes our respondents reported in regards to EBP did not result in greater use of clinical practice guidelines remains unclear. A systematic review of barriers to clinical practice guideline adherence identified six main issues: lack of awareness about the availability of guidelines; lack of agreement with the interpretation or applicability of guidelines; perceived inability to implement guidelines; guideline recommendations perceived as ineffective; inertia of previous practice; and external barriers such as patient-related barriers like patients not accepting guideline recommendations, or environmental related factors such as inadequate resources [44]. To what extent each issue influenced our respondents use of clinical practice guidelines is uncertain as barriers depend on the healthcare setting, patient population, and content of guidelines [44]. Therefore, we recommend that if further studies are conducted that they identify barriers particular to the use of clinical guidelines in chiropractic practice However, studies examining educational interventions to increase clinical guideline adherence have found that content addressing barriers tends to result in little change unless drivers of cultural change are also incorporated [45]. For instance, educational interventions that improved primary care physicians’ knowledge about cholesterol lowering therapy did not affect prescribing behaviour unless the educational material was presented by a respected secondary care specialist. Hence, studies that gather information about barriers to clinical guideline use in chiropractic practice need also examine the cultural factors that promote behaviour change.

An approach that could be useful to improve chiropractors’ uptake of clinical practice guidelines is the use of software that integrates electronic patient files with clinical decision making rules. At present, no such software exists within the field of manual therapy, but it could be developed by including algorithms based on the results of several studies that have identified subgroups of patients who respond better to particular manual therapy treatments [46-51]. Electronic evidence-based clinical advice has led to increased clinician adherence to clinical practice guidelines in other healthcare professions, and the development and implementation of electronic advice in chiropractic practice could result in similar changes in clinical behaviour [52-54].

We used a rigorous approach to evaluating the structure of the Jette et al. questionnaire, consisting of an assessment of content validity, dimensionality, and internal consistency. The questionnaire had originally been developed to survey physiotherapists, but our modifications render it more suitable for studies of chiropractors. Hence, we recommend the use of our adapted questionnaire in future studies of EBP use among chiropractors. Such studies could also consider assessing the test-retest reliability of the question to further consolidate its psychometric properties.

### Limitations

Most respondents stated that they read research literature, but the survey instrument did not capture information regarding the quality of the research literature. As such, it is unclear if the articles were sound scientific papers or opinion based commentaries. Hence, any further studies should evaluate the quality of literature chiropractors are reading. Although we know the total number of registered chiropractors in Australia we are unable to calculate the response rate to this survey because we relied on third parties to distribute the questionnaire via email so therefore we do not know how many chiropractors actually received the invitation to participate. Even so, the number of returned questionnaires (n = 584) was low, given that the questionnaire may potentially have been distributed to all those registered (n = 4378). The low number of responses may have in part resulted from the Chiropractors Board of Australia invitation notice being embedded in their newsletter and hence being lost amongst the other information. However, our sample size is reasonable and the generalisability of our findings is partially supported by the similarities in demographic characteristics between our survey respondents and demographic material made available by the Chiropractic Board of Australia. But the Chiropractic Board of Australia data was limited to age and location and it is likely that our respondents held more favourable perceptions about EBP than non-respondents (volunteer bias). Considered together, this suggests that caution is needed in determining whether the views of our sample towards EBP would be representative of all Australian chiropractors.

## Conclusion

This study found that many Australian chiropractors generally hold positive attitudes towards EBP, thought EBP was useful, and were interested in improving EBP skills. Despite the favourable inclination towards EBP, most Australian chiropractors did not use clinical practice guidelines. The reasons behind the low rate of clinical practice guideline use were unclear and further studies are warranted to examine barriers to guideline use. Importantly, chiropractors were much more likely to use clinical practice guidelines when guidelines were perceived as aiding decisions about patient care. This suggests that tertiary educational institutions and continuing professional development programs should highlight how the use of clinical practice guidelines can enhance clinical decision making and result in improved patient outcomes.

## Abbreviations

EBP: Evidence-based practice; CVI: Content validity index; CBA: Chiropractors” Board of Australia.

## Competing interests

JH, SF and BW are Board members of the Chiropractic and Osteopathic College of Australasia Research Limited, which provided funding for this project but had no other role in the project conduct or reporting of results. BW, SF, and NS are on the editorial team of *Chiropractic and Manual Therapies* but had no involvement in the editorial process of this manuscript.

## Authors’ contributions

All authors contributed to the conception and design of the study, acquisition of data, analysis and interpretation of data, were involved in drafting the manuscript, and have given final approval of the version to be published.

## Supplementary Material

Additional file 1 The adapted questionnaire. Click here for file
