# Supplementary material for: A survey of Australian chiropractors’ attitudes and beliefs about evidence-based practice and their use of research literature and clinical practice guidelines
Source: Chiropr Man Therap. 2013 Dec 17;21:44. doi: 10.1186/2045-709X-21-44 (PMC3878410; doi:10.1186/2045-709X-21-44)
Supplement: Additional file 1 — The adapted questionnaire. [file 2045-709X-21-44-S1.docx]

**Additional file 1: The adapted questionnaire**

| item | statement |
| --- | --- |
| 1 | Application of EBP is necessary in the practice of chiropractic |
| 2 | Literature and research findings are useful in my day-to-day practice |
| 3 | The adoption of EBP places an unreasonable demand on chiropractors |
| 4 | I am interested in learning or improving the skills necessary to incorporate EBP into my practice. |
| 5 | EBP improves the quality of patient care. |
| 6 | Strong evidence is lacking to support most of the interventions I use with my patients |
| 7 | EBP helps me make decisions about patient care |
| 8 | Read/review research/literature related to my clinical practice |
| 9 | I use professional literature and research findings in the process of clinical decision-making |
| 10 | I use The Cochrane Library, MEDLINE, PUBMED or other databases to search for practice-relevant literature/research |
| 11 | Practice guidelines are available for topics related to my practice |
| 12 | I actively seek practice guidelines pertaining to areas of my practice |
| 13 | I use practice guidelines in my practice |
| 14 | I am aware that practice guidelines are available online |
| 15 | I am able to access practice guidelines online |
| 16 | I am able to incorporate patient preferences with practice guidelines |
| 17 | I have access to current research through professional journals in their paper form or on the Internet |
| 18 | I have the ability to access relevant databases and the Internet at my practice |
| 19 | I have the ability to access relevant databases and the Internet at home or locations other than my practice |
| 20 | I learned the foundations for EBP as part of my undergraduate chiropractic education |
| 21 | I have received formal training in search strategies for finding research relevant to my practice |
| 22 | I am familiar with the medical research databases (eg, MEDLINE, PUBMED, CINAHL, The Cochrane Library) |
| 23 | I received formal training in critical appraisal of research literature as part of my undergraduate chiropractic education |
| 24 | I am confident in my ability to critically review professional literature |
| 25 | I am confident in my ability to find relevant research to answer my clinical questions |
| 26 | Please specify your three top barriers to the use of EBP in your clinical practice |
| 27 | Please specify the 3 most common sources of the research evidence that you utilise in your chiropractic practice |
| 28 | Are you a currently registered Australian chiropractor |
| 29 | What is your sex |
| 30 | What is your age |
| 31 | I am a member of the: Chiropractic Association of Australia; Chiropractic and osteopathic College of Australasia; Australian Spinal Research Foundation |
| 32 | For how many years have you been registered as a chiropractor |
| 33 | Do you regularly (at least once per year) participate in continuing education courses |
| 34 | On average, how many hours per week do you work |
| 35 | On average, how many patients do you see on a typical day in practice |
| 36 | Which of the following best describes the location of the practice in which you perform the majority of your patient care |
| 37 | Indicate which of the following states or territories in which you practice (mark all that apply) |
| 38 | EBP is defined as the “integration of best research evidence with clinical expertise and patient values and circumstances” (Straus et al. 2011). Do you think anything is missing from this definition for chiropractic practice? Please comment below. |
| 39 | Please provide any comments you would like to the investigators of this study |
